# Identification of genes associated with methotrexate resistance in methotrexate-resistant osteosarcoma cell lines

**DOI:** 10.1186/s13018-015-0275-8

**Published:** 2015-09-04

**Authors:** Xiao-rong Yang, Yan Xiong, Hong Duan, Ren-rong Gong

**Affiliations:** Department of Operation Room, West China Hospital, Sichuan University, No 37, Guo Xue Lane, Chengdu, Sichuan 610041 People’s Republic of China; Department of Orthopedics, West China Hospital, Sichuan University, No 37, Guo Xue Lane, Chengdu, Sichuan 610041 People’s Republic of China

**Keywords:** Osteosarcoma, Methotrexate resistance, Microarray data

## Abstract

**Background:**

This study aimed to better understand the mechanisms underlying methotrexate (MTX)—resistance in osteosarcoma.

**Methods:**

The raw transcription microarray data GSE16089 collected from three MTX-sensitive osteosarcoma (Saos-2) cell samples and three MTX-resistant osteosarcoma (Saos-2) cell samples were downloaded from Gene Expression Omnibus. After data processing, the differentially expressed genes (DEGs) were identified. Next, DEGs were submitted to DAVID for functional annotation based on the GO (Gene Ontology) database, as well as pathway enrichment analysis based on the KEGG (Kyoto Encyclopedia of Genes and Genomes) database. Transcription factors (TFs) and tumor-associated genes (TAGs) were identified with reference to TRANSFAC and TAG, and TSGene databases, respectively. The protein-protein interaction (PPI) network of the gene-encoded products was constructed, and the subnetwork with the highest score was also detected using Search Tool for the Retrieval of Interacting Genes and BioNet package.

**Results:**

A total of 690 up-regulated genes and down-regulated 626 genes were identified. Up-regulated DEGs (including *AARS* and *PARS2*) were associated to transfer RNA (tRNA) aminoacylation while down-regulated DEGs (including *AURKA*, *CCNB1*, *CCNE2*, *CDK1*, and *CENPA*) were correlated with mitotic cell cycle. Totally, 13 TFs (including *HMGB2*), 13 oncogenes (including *CCNA2* and *AURKA*), and 19 tumor suppressor genes (TSGs) (including *CDKN2C*) were identified from the down-regulated DEGs. Ten DEGs, including nine down-regulated genes (such as *AURKA*, *CDK1*, *CCNE2*, and *CENPA*) and one up-regulated gene (*GADD45A*), were involved in the highest score subnetwork.

**Conclusion:**

*AARS*, *AURKA*, *AURKB*, *CENPA*, *CCNB1*, *CCNE2*, and *CDK* may contribute to MTX resistance via aminoacyl-tRNA biosynthesis pathway, cell cycle pathway, or p53 signaling pathway.

## Background

Methotrexate (MTX) was first introduced to replace aminopterin to treat acute lymphocytic leukemia, which works via inhibiting dihydrofolate reductase (DHFR), a key enzyme required in intracellular folate metabolism, leading to decreased tretrahydrofolate coenzyme level, accordingly achieving the inhibition of thymidylate and the biosynthesis of DNA and purine. So far, different mechanisms have been presented to address the intrinsic and acquired MTX resistance: (1) decreased MTX transport, (2) impaired MTX polyglutamylation, (3) increased DHFR enzyme activity, (4) altered affinity of MTX for DHFR, and (5) increased MTX efflux due to elevated levels of the multidrug resistance protein (MRP) [1].

Osteosarcoma is a primary malignant, highly vascularized bone tumor, mainly occurring in adolescents and children [2, 3]. The unclear understanding of the underlying molecular mechanism greatly hinders the therapy of osteosarcoma [4]. Currently, multiagent chemotherapy, usually using doxorubicin, cisplatin, and high-dose MTX, has improved the survival of osteosarcoma patients from 11 to 70 % [5]. However, MTX resistance has become an issue of growing interest, as little information is available in this disease up to now [6]. *TP53*, a tumor suppressor gene (TSG), encodes a transcriptional regulator that responds to DNA damage or cellular stress and controls the progression and apoptosis of cell cycle. As previously reported, the accumulation of p53 protein is probably a predictor of response to methotrexate (MTX) [7]. p53 alterations increase the risk for the development of drug resistance by altering MTX transport [8].

Using the transcription profiles of three MTX-sensitive osteosarcoma (Saos-2) cell lines and three MTX-resistant Saos-2 cell lines and analyzing the network of the differentially expressed genes (DEGs), Selga et al. have found the alteration in the expression of a number of genes, such as eukaryotic translation elongation factor 1 alpha 1 (*EEF1A1*) in the MTX-resistant osteosarcomas (Saos-2) cell lines, pancreatic cancer, and erythroblastic leukemia cell lines [9]. However, further systematic analyses, including GO (Gene Ontology) functional and REACTOME pathway enrichment analysis, were not performed for DEGs concerning osteosarcoma cells in their study.

REACTOME is a knowledgebase of human reactions and pathways, which provides an integrated view of the molecular details of human biological processes ranging from metabolism to DNA replication and repair to signaling cascades [10], and has been used in various studies [11]. The transcription microarray data GSE16089 deposited in Gene Expression Omnibus (GEO), which includes three chips from MTX-sensitive and three from MTX-resistant osteosarcoma (Saos-2) cell lines [9], were downloaded and analyzed in this study so as to better understand the genetic etiology of osteosarcoma. The DEGs were identified, and the functional and pathway enrichment analysis was performed for them. The protein-protein interaction (PPI) network of the gene productions and its subnetwork were analyzed. These findings in this study will encourage us to investigate the anti-cancer effects of the DEGs or the pathways as well as the MTX resistance in osteosarcoma.

## Material and methods

As the study did not involve any human or animal study, the ethical approval was not required.

### Microarray data

Gene expression microarray dataset deposited in the National Center of Biotechnology Information (NCBI) GEO (http://www.ncbi.nlm.nih.gov/geo/) with the accession number of GSE16089 was downloaded [9]. The annotation platform was GPL570 [HG-U133_Plus_2] Affymetrix Human Genome U133 Plus 2.0 Array. According to contributors, three samples of either MTX-sensitive cells or MTX-resistant cells of the Saos-2 osteosarcoma cell line were used for gene expression analyses [9]. Saos-2 cell line was sensitive to MTX, and its MTX-resistant cells were obtained in the laboratory via incubation with stepwise concentrations of MTX (Lederle) as described previously [12].

### Data processing

The raw probe profile data was downloaded from GEO. The processing of the raw microarray data was performed by robust multiarray average (RMA) using R/Bioconductor package Affy [13]. The preprocessing consisted of background correction, quantile normalization, and probe summarization of expression value. The gene expression matrixes were obtained for further analysis.

### Identification of DEGs

Transcriptional sets were mapped to NCBI entrez genes using Gene ID converter [14]. The averaged value was calculated for further analysis if there were multiple probe sets corresponding to the same gene. Probes were filtered if they corresponded to multiple genes. The classical *t* test was performed among the samples to identify the genes specifically differentially expressed between MTX-sensitive and MTX-resistant Saos-2 cell lines. The cut-off criteria for the DEGs were set at *p* value <0.05 and |log2FC (fold change)| > 1.

### Functional and pathway enrichment analysis

GO and KEGG (Kyoto Encyclopedia of Genes and Genomes) pathway analysis provides prediction of gene function and informs people of how molecules or genes work [15, 16]. The DEGs were submitted to DAVID (Database for Annotation, Visualization, and Integrated Discovery) (http://david.abcc.ncifcrf.gov/) to find the significantly enriched biological process (BP) terms, molecular function (MF) terms, and cellular component (CC) terms based on the GO (Gene Ontology) database, as well as pathways based on the KEGG (Kyoto Encyclopedia of Genes and Genomes) database. For the identification of the significantly enriched biological processes in detail, the significantly altered DEGs were subjected to the REACTOME knowledgebase (http://www.reactome.org). The thresholds for the significant associated GO functional category and pathways were set at *p* value < 0.01.

### Identification of transcription factors and tumor-associated genes

TRANSFAC (http://www.gene-regulation.com/index2) is a database on transcription factors, their genomic binding sites, and DNA-binding profiles [17]. To identify the DEGs that also act as transcription factors, transcription factor (TF) prediction was performed using the TRANSFAC database. TAG (tumor-associated gene) database (http://www.binfo.ncku.edu.tw/TAG/) is a semi-automatic information retrieving engine which collects specific information about genes from various resources. TSGene database (http://bioinfo.mc.vanderbilt.edu/TSGene/) is a resource of tumor suppressor genes (TSGs) that provides a comprehensive TSG catalog for advanced systems biology-based analysis for the cancer research community [18]. TAGs including oncogenes and TSGs were also identified from the DEGs using TAG and TSGene databases, respectively.

### Construction of PPI network and the subnetwork analysis

STRING (Search Tool for the Retrieval of Interacting Genes) is a web server to retrieve and display the repeatedly occurring neighborhood of a gene which generalizes access to protein interaction data, by integrating known and predicted interactions from a variety of sources [19]. To describe the interactive network of DEGs, the STRING database was used to build the PPI network of encoding products of all of the DEGs. A STRING score of 0.4 was set as the reliability threshold. Cytoscape, a standard tool for integrated analysis and visualization of biological networks, was used to visualize the PPI network [20]. The connectivity degree analysis was performed, and hub nodes were obtained using the scale-free properties of PPI networks. The BioNet package is an R-Package for the functional analysis of biological networks and is used for the mining of the sub-networks in the PPI network [21]. The highest scoring subnetwork was obtained. The threshold of the given false discovery rate (FDR) value was 0.0001.

## Results

### Identification of DEGs

After data processing, a total of 4461 transcripts that were differentially expressed between MTX-sensitive and MTX-resistant Saos-2 cell lines were identified, 2300 up-regulated and 2161 down-regulated transcripts. Finally, 1316 DEGs were obtained, including 690 up-regulated DEGs (e.g., *AARS*, *TARS*, *YARS*, *CCND1*, *PARS2*, and *GADD45A*) and 626 down-regulated DEGs (e.g., *AURKA*, *AURKB*, *CCNB1*, *CDK1*, *CDKN2C*, *CENPA*, and *HMGB2*).

### Functional and pathway enrichment analysis

According to the GO annotation, the up-regulated DEGs were functionally involved in BP terms such as response to endoplasmic reticulum stress (including *AARS* and *CCND1*) and transfer RNA (tRNA) aminoacylation for protein translation (including *AARS*, *TARS*, *YARS*, and *PARS2*), and MF terms such as aminoacyl-tRNA ligase activity (including *AARS* and *PARS2*), as well as CC terms such as intracellular membrane-bounded organelle (including *CCND1* and *GADD45A*) (Table 1). Moreover, the up-regulated DEGs were mainly associated with the KEGG pathways such as aminoacyl-tRNA biosynthesis (including *AARS* and *PARS2*). The mainly related REACTOME pathways were cytosolic tRNA aminoacylation (including *AARS*) and tRNA aminoacylation (including *AARS* and *PARS2*) (Table 2).

**Table 1:** The top five terms of Gene Ontology category analysis for the up-regulated differentially expressed genes

Category	Term	Name	Counts	Gene symbol	*p* value
Biological process	GO:0034976	Response to endoplasmic reticulum stress	21	*AARS*, *CCND1*, *FAM129A*, *GSK3B*, …	3.17E−10
	GO:0006418	tRNA aminoacylation for protein translation	12	*AARS*, *PARS2*, *TARS*, *YARS*, …	3.41E−08
	GO:0043038	Amino acid activation	12	*AARS*, *PARS2*, *TARS*, *YARS*, …	7.23E−08
	GO:0043039	tRNA aminoacylation	12	*AARS*, *PARS2*, *TARS*, *YARS*, …	7.23E−08
	GO:0034620	Cellular response to unfolded protein	14	*AARS*, *CCND1*, *SERP1*, *STC2*, …	1.35E−06
Molecular function	GO:0004812	Aminoacyl-tRNA ligase activity	12	*AARS*, *PARS2*, *TARS*, *YARS*, …	6.72E−09
	GO:0016875	Ligase activity, forming carbon-oxygen bonds	12	*AARS*, *PARS2*, *TARS*, *YARS*, …	6.72E−09
	GO:0016876	Ligase activity, forming aminoacyl-tRNA and related compounds	12	*AARS*, *PARS2*, *TARS*, *YARS*, …	6.72E−09
	GO:0015175	Neutral amino acid transmembrane transporter activity	7	*SLC1A4*, *SLC1A5*, *SLC3A2*, *SLC7A5*, …	2.70E−06
	GO:0015171	Amino acid transmembrane transporter activity	9	*SLC1A4*, *SLC1A5*, *SLC3A2*, *SLC7A5*, …	1.74E−4
Cellular components	GO:0043231	Intracellular membrane-bounded organelle	370	*CCND1*, *COL1A2*, *EEF1A2*, *GADD45A*, …	5.07E−06
	GO:0043227	Membrane-bounded organelle	371	*CCND1*, *COL1A2*, *EEF1A2*, *GADD45A*, …	5.19E−06
	GO:0005622	Intracellular	448	*AARS*, *CCND1*, *EEF1A2*, *GADD45A*, …	3.09E−05
	GO:0044424	Intracellular part	443	*AARS*, *CCND1*, *EEF1A2*, *GADD45A*, …	4.49E−05
	GO:0005737	Cytoplasm	346	*AARS*, *CD9*, *CCND1*, *EEF1A2*, …	8.23E−05

**Table 2:** Pathway enrichment of the up-regulated differentially expressed genes

Pathway	Term	Name	Count	Gene symbol	*p* value
KEGG pathway	970	Aminoacyl-tRNA biosynthesis	12	*AARS*, *PARS2*, *TARS*, *YARS*, …	6.95E−07
	260	Glycine, serine, and threonine metabolism	5	*CBS*, *CTH*, *PHGDH*, *PSAT1*, *SHMT2*	3.5E−03
	532	Glycosaminoglycan biosynthesis-chondroitin sulfate	4	*CHPF*, *CHST15*, *UST*, *XYLT1*	5.2E−03
	565	Ether lipid metabolism	5	*ENPP2*, *LPCAT1*, *PLA2G12A*, *PLA2G4A*, *PPAP2B*	6.0E−03
REACTOME pathway (top ten terms)	379716	Cytosolic tRNA aminoacylation	10	*AARS*, *PARS2*, *TARS*, *YARS*, …	1.50E−09
	379724	tRNA aminoacylation	12	*AARS*, *PARS2*, *TARS*, *YARS*, …	5.17E−09
	352230	Amino acid transport across the plasma membrane	8	*SLC1A4*, *SLC1A5*, *SLC3A2*, *SLC7A5*, …	4.75E−06
	425374	Amino acid and oligopeptide SLC transporters	9	*SLC1A4*, *SLC1A5*, *SLC3A2*, *SLC7A5*, …	2.39E−05
	380994	Activation of genes by ATF4	4	*ASNS*, *ATF3*, *DDIT3*, *HERPUD1*	3.61E−05
	70614	Amino acid synthesis and interconversion (transamination)	5	*ASNS*, *GLS*, *GPT2*, *PHGDH*, *PSAT1*	0.000160459
	381042	PERK regulated gene expression	4	*ASNS*, *ATF3*, *DDIT3*, *HERPUD1*	4.48E−04
	1614603	Cysteine formation from homocysteine	2	*CBS*, *CTH*	1.1E−03
	73943	Reversal of alkylation damage by DNA dioxygenases	2	*ALKBH2*, *ALKBH3*	1.1E−03
	73942	DNA damage reversal	2	*ALKBH2*, *ALKBH3*	3.1E−03

*KEGG* Kyoto Encyclopedia of Genes and Genomes

The down-regulated DEGs were significantly enriched in BP terms such as microtubule cytoskeleton organization and mitotic cell cycle (including *AURKA*, *AURKB*, *CCNB1*, *CDK1*, and *CENPA*) and in MF terms such as protein binding and nucleotide binding (including *AURKA*, *AURKB*, *CCNB1*, *CDK1*, and *CENPA*), as well as in CC terms related to chromosome, centromeric region (including *AURKB*, *CCNB1*, *CENPA*, and *CENPH*), and kinetochore (including *CCNB1*, *CENPA*, and *CENPH*) (Table 3). Also, the down-regulated DEGs were significantly involved in KEGG pathways of cell cycle, oocyte meiosis, and p53 signaling pathway (including *CCNB1*, *CCNE2*, and *CDK1*). The relevant REACTOME pathways were resolution of sister chromatid cohesion and mitotic M-M/G1 phases (including *AURKB*, *CCNB1*, *CDK1*, *CENPA*, and *CENPH*) (Table 4).

**Table 3:** The top five terms of Gene Ontology category analysis for the down-regulated differentially expressed genes

Category	Term	Name	Counts	Gene symbol	*p* value
Biological process	GO:0000226	Microtubule cytoskeleton organization	46	*AURKA*, *AURKB*, *CCNB1 CDK1*, *CENPA*, *FBXO5*, …	0
	GO:0000278	Mitotic cell cycle	119	*AURKA*, *AURKB*, *CCNE2*, *CDK1*, *CENPA*, *CENPH*, *FBXO5*, *RRM2*, …	0
	GO:0000280	Nuclear division	68	*AURKA*, *AURKB*, *CCNB1*, *CCNB2*, *CENPE*, …	0
	GO:0006996	Organelle organization	164	*AURKA*, *AURKB*, *CCNB1*, *CCNB2*, *CDK1*, *CENPA*, *CENPE*, *CENPH*, …	0
	GO:0007017	Microtubule-based process	59	*AURKA*, *AURKB*, *CCNB1*, *CDK1*, *CENPA*, *FBXO5*, …	0
Molecular function	GO:0005515	Protein binding	334	*AURKA*, *AURKB*, *CCNB1*, *CCNB2*, *CCNE2*, *CDK1*, *HMGB2*, …	8.15E−13
	GO:0000166	Nucleotide binding	122	*AURKA*, *AURKB*, *CDK1*, *CENPE*, *EEF1A1*, …	1.32E−07
	GO:1901265	Nucleoside phosphate binding	122	*AURKA*, *AURKB*, *CDK1*, *CENPE*, *EEF1A1*, …	1.35E−07
	GO:0036094	Small molecule binding	127	*AURKA*, *AURKB*, *CDK1*, *CENPE*, *EEF1A1*, …	2.24E−07
	GO:0008017	Microtubule binding	19	*CENPE*, *KIF14*, *KIF15*, *PLK1*, …	8.25E−07
Cellular components	GO:0000775	Chromosome, centromeric region	37	*AURKB*, *BUB1*, *CCNB1*, *CENPA*, *CENPH*, …	0
	GO:0000776	Kinetochore	29	*CCNB1*, *CENPA*, *CENPE CENPH*, *KIF22*, …	0
	GO:0000777	Condensed chromosome kinetochore	27	*CCNB1*, *CENPA*, *CENPE CENPH*, *KIF2C*, …	0
	GO:0000779	Condensed chromosome, centromeric region	28	*AURKB*, *CCNB1*, *CENPA*, *CENPE*, *CENPH*, …	0
	GO:0000793	Condensed chromosome	42	*AURKB*, *CCNB1*, CE*N*PA, *CENPH*, *KIF2C*, …	0

**Table 4:** Pathway enrichment analysis of the down-regulated differentially expressed genes

Pathway	Term	Name	Count	Gene symbol	*p* value
KEGG pathway	4110	Cell cycle	21	*CCNA2*, *CCNB1*, *CCNB2*, *CCNE2*, *CDK1*, *CDKN2C*, …	2.35E−09
	5322	Systemic lupus erythematosus	15	*H2AFX*, *H2AFY*, *HIST1H2AE*, *HIST1H2AM*, …	1.07E−04
	4114	Oocyte meiosis	13	*AURKA*, *CCNB1*, *CCNB2*, *CCNE2*, *CDK1*, *FBXO5*, …	1.86E−04
	4115	p53 signaling pathway	9	*CCNB1*, *CCNB2*, *CCNE2*, *CDK1*, *GADD45G*, …	7.05E−04
	30	Pentose phosphate pathway	5	*ALDOC*, *DERA*, *PFKP*, *PGM1*, *PRPS1*	2.5E−03
	4914	Progesterone-mediated oocyte maturation	9	*CCNA2*, *CCNB1*, *CCNB2*, *CDK1*, *PLK1*, …	3.7E−03
	3030	DNA replication	5	*FEN1*, *LIG1*, *POLA2*, *POLE*, *POLE2*	9.0E−03
REACTOME pathway (top ten terms)	1640170	Cell cycle	90	*AURKA*, *AURKB*, *CCNB1*, *CCNE2*, *CDK1*, *CENPA*, *CENPH*, …	0
	2500257	Resolution of sister chromatid cohesion	34	*AURKB*, *CCNB1*, *CCNB2*, *CDK1*, *CENPA*, *CENPH*, …	0
	453277	Mitotic M-M/G1 phases	57	*AURKB*, *CCNB1*, *CCNB2*, *CDK1*, *CENPA*, *CENPH*, …	0
	68877	Mitotic prometaphase	39	*AURKB*, *CCNB1*, *CCNB2*, *CDK1*, *CENPA*, *CENPH*, …	0
	68886	M phase	48	*AURKB*, *CCNB1*, *CCNB2*, *CDK1*, *CENPA*, *CENPH*, …	0
	69278	Cell cycle, mitotic	79	*AURKA*, *AURKB*, *CCNB1*, *CCNE2*, *CDK1*, *CENPA*, *CENPH*, …	0
	2555396	Mitotic metaphase and anaphase	38	*AURKB*, *CENPA*, *CENPH*, *FBXO5*, *PLK1*, *PSMD1*, …	1.11E−16
	2467813	Separation of sister chromatids	36	*AURKB*, *CENPA*, *CENPH*, *PLK1*, *PSMD1*, …	4.44E−16
	68882	Mitotic anaphase	37	*AURKB*, *CENPA*, *CENPH*, *KIF2C*, *PSMD1*, …	4.44E−16
	69275	G2/M transition	23	*AURKA*, *CCNA2*, *CCNB1*, *CCNB2*, *CDK1*, *PLK1*, *PLK4*, …	6.87E−10

*KEGG* Kyoto Encyclopedia of Genes and Genomes

### Identification of TFs, oncogenes, and TSGs

After the annotation, 13 TFs (*TAF9*, *RUNX3*, *RUNX2*, *PBX1*, *MYBL1*, *MSX2*, *MEIS2*, *HMGB2*, *GTF2H2*, *FOXM1*, *FOXD1*, *EZH2*, and *BRIP1*), 13 oncogenes (*WHSC1*, *SERTAD1*, *RUNX2*, *RBM3*, *PTTG1*, *PBX1*, *MYBL1*, *MLLT11*, *KIT*, *HMMR*, *CEP55*, *CCNA2*, and *AURKA*), and 19 TSGs (*TGFBI*, *S100A2*, *RUNX3*, *PPP1R1B*, *PLK2*, *MSH2*, *ING3*, *IGFBP7*, *IGFBP3*, *HTRA1*, *GADD45G*, *FANCG*, *EGLN3*, *EEF1A1*, *CLU*, *CDKN2C*, *CDH13*, *CCDC136*, *BUB1B*) were identified. However, no TFs, oncogenes, or TSGs were identified from the up-regulated DEGs.

### PPI network and the subnetwork analysis

**Fig. 1:**
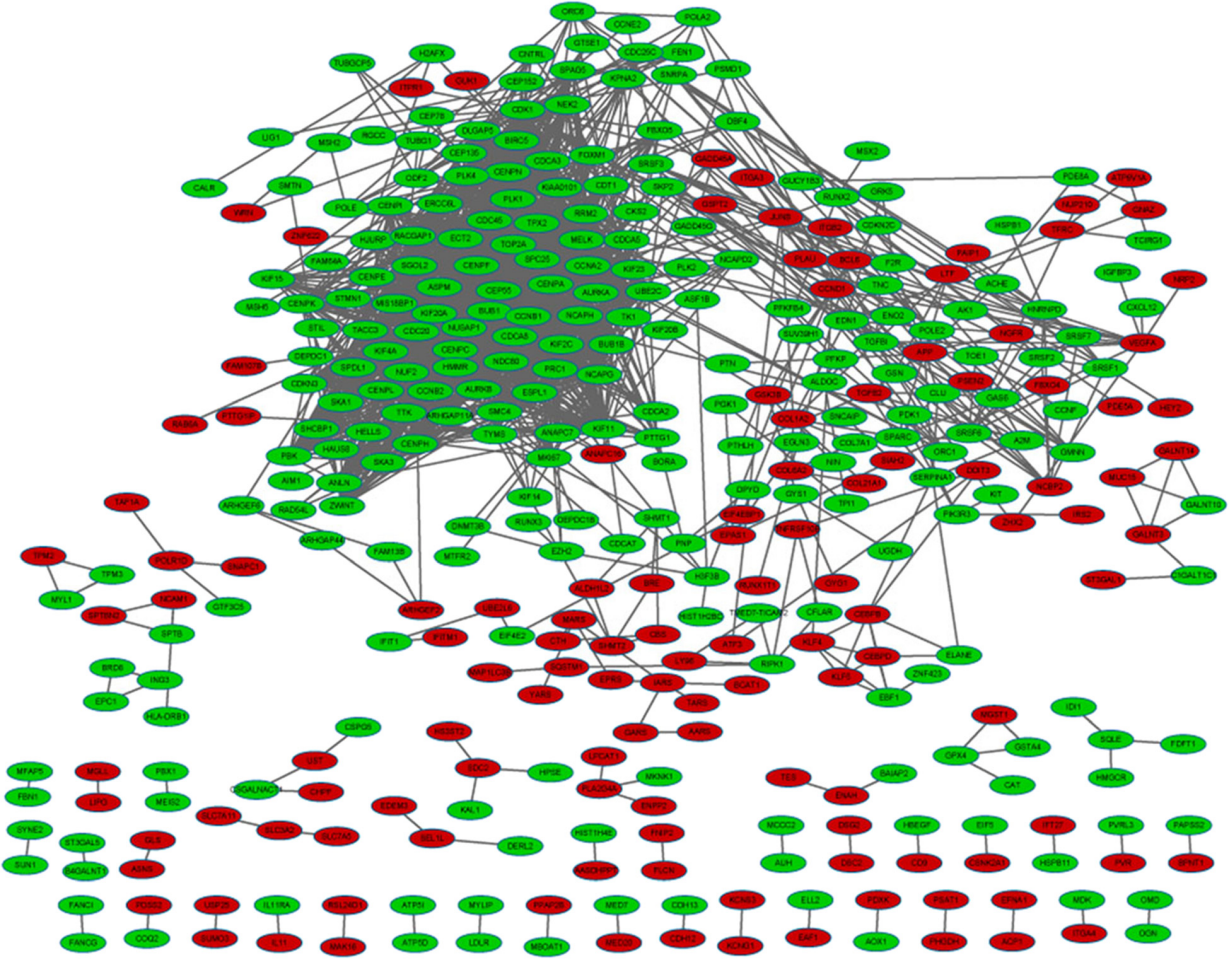
The protein-protein interaction network for the differentially expressed genes (DEGs). The gene productions are indicated by *ellipse dots* and the linkages among them are indicated by *edges. Red* stands for the productions of up-regulated DEGs and *green* for productions of down-regulated DEGs

**Fig. 2:**
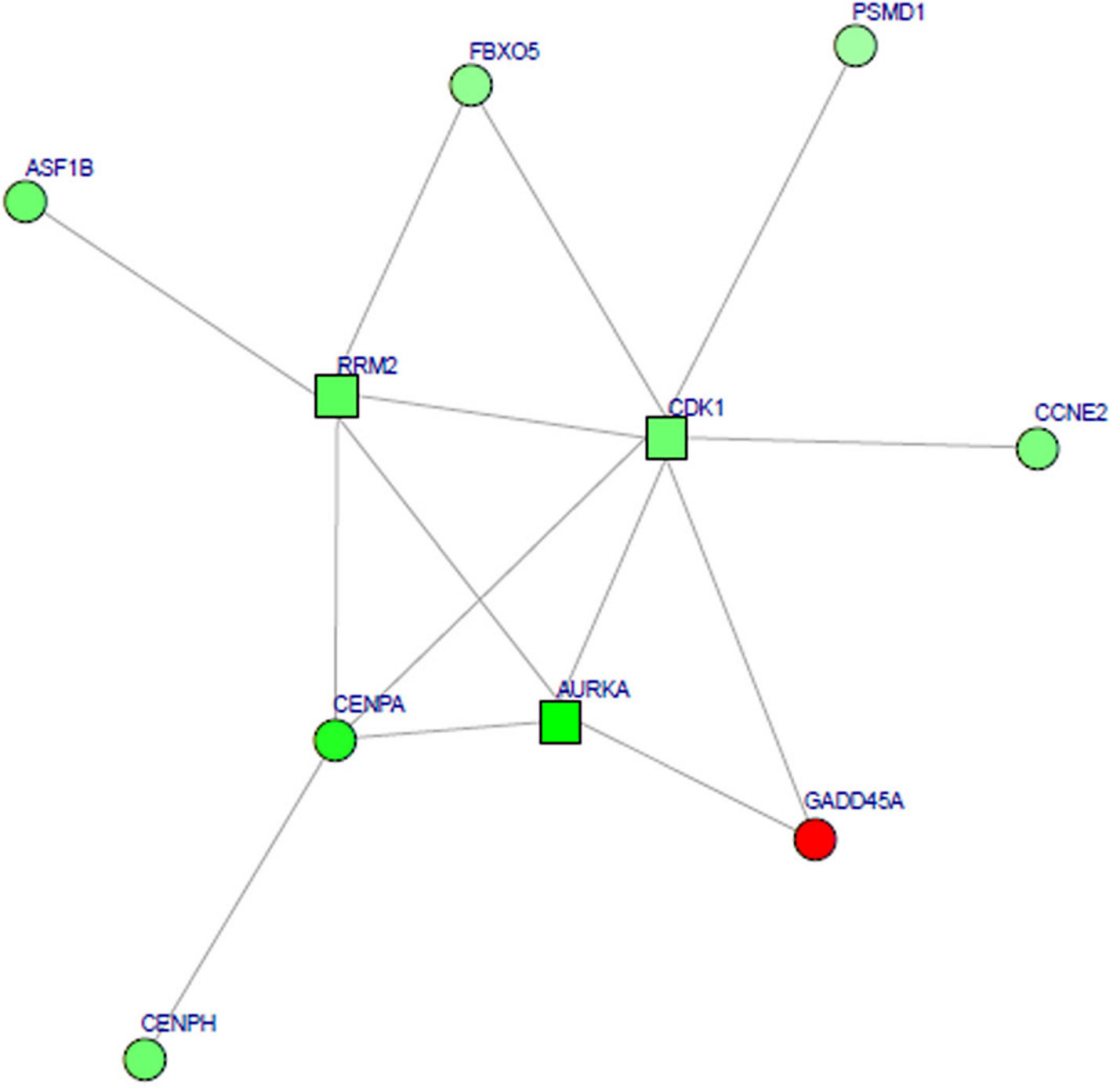
The highest score subnetwork for the differentially expressed genes (DEGs). *Red* stands for the productions of up-regulated DEGs and *green* for productions of down-regulated DEGs. *Round dot* indicates the highly associated DEGs with osteosarcoma and *square* indicates less relevant DEGs

After construction and visualization for the PPI network of the DEG products, a total of 375 nodes were included, such as *CDK1* (degree = 82), *CCNA2* (degree = 64), *CCNB1* (degree = 62), and *CENPE* (degree = 56) (Fig. 1). The subnetwork with the highest score contained ten gene-encoding proteins, namely *CDK1*, *RRM2*, *CENPA*, *AURKA*, *FBXO5*, *CCNE2*, *CENPH*, *ASF1B*, *PSMD1*, and *GADD45A* (Fig. 2).

## Discussion

In this study, a total of 690 up-regulated DEGs and 626 down-regulated DEGs were identified in MTX-resistant osteosarcoma cells. According to the functional and pathway enrichment analysis, the up-regulated DEGs such as *AARS*, *TARS*, *YARS*, and *PARS2* were mainly associated with tRNA aminoacylation, and the down-regulated DEGs such as *AURKA*, *AURKB*, *CCNB1*, *CDK1*, and *CENPA* were mainly correlated with mitotic cell cycle.

The up-regulated DEGs, including *AARS*, *TARS*, *YARS*, and *PARS2*, were biologically related to tRNA aminoacylation. *AARS*, *TARS*, and *YARS* encode aminoacyl-tRNA synthetases alanyl-tRNA synthetase, threonyl-tRNA synthetase, and tyrosyl-tRNA synthetase, respectively, which catalyze the aminoacylation of tRNA by their cognate amino acid, and thus are necessary for protein synthesis. Aminoacyl-tRNA synthetases usually take distinct roles in inflammation and transcriptional regulation [22]. The aberrant expression and cellular localization of aminoacyl-tRNA synthetases disturb normal cell regulatory networks and cause abnormalities through multiple routes [23]. For example, inhibition of osteosarcoma cell migration might be related to the extracellular functions of *TARS* [24]. MTX has been used to treat antisynthetase syndrome, a type of heterogeneous autoimmune disorder, in which autoantibodies target anti-aminoacyl-transfer RNA synthetase for specific amino acid. The up-regulation of aminoacyl-tRNA synthetases in MTX-resistant cells may indicate that this type of cells has developed an ability to improve the expression level of aminoacyl-tRNA synthetases in the presence of MTX that offset the effect of MTX, leading to MTX resistance.

The down-regulated genes were dominantly related to mitotic cell cycle or nuclear division, for example, *AURKA*, *AURKB*, *CENPA*, *CDK1*, *CCNB1*, and *CCNB2. AURKA* is known as an oncogene. AURKA is an important regulator to G2/M transition [25]. AURKA protein is mainly located at the microtubule organizing center at the metaphase I (M1) of oocytes [26]. Further investigations have shown that the localization of AURKA in the area of aligned chromosomes is consistent with the AURKA-dependent phosphorylation of kinetochore component centromere protein A (CENPA). CENPA phosphorylation requires the enrichment of AURKB to maintain the phosphorylation on Ser7 at inner centromeres and for kinetochore function [26–28]. Jiang et al. [29] showed that silencing of *AURKA* expression in osteosarcoma cells significantly decreased both colony formation ability in vitro and tumorigenesis ability in vivo as well as induced cell apoptosis and G2/M cell cycle arrest in osteosarcoma cells. In addition, the median survival time was significantly longer in patients with low-*CENPA* expression osteosarcomas than in those with high-*CENPA* expression osteosarcomas. *AURKA* and *CENPA* had, respectively, been identified as a susceptibility gene and an independent poor prognostic factor for osteosarcoma [25, 30]. The down-regulation of mitosis-related genes seems contrary to the commonly known fact that mitosis-related genes are usually up-regulated in tumors. However, up-regulation of mitosis-related genes is just recognized in tumors, not in drug-resistant tumor cells. Actually, we know little about the anti-drug mechanisms in tumors. It is possible that down-regulation of mitosis-related genes is a protection mechanism in MTX-resistant cells, or just at certain time point. Apparently, this finding has to be validated experimentally, since the sample number is so small.

Furthermore, *CDK1*, *CCNB1*, and *CCNB2* which were enriched in mitotic cell cycle, oocyte meiosis, and p53 signaling pathways were inactivated by MTX treatment in osteosarcoma cell lines. As we know, deregulated cell proliferation and tumor-associated cell cycle always propel the complexity and idiopathy of cancer [31, 32]. Tumor-associated cell cycle is often mediated by the alterations of cyclin-dependent kinase (CDK) activities [33]. Cyclin B1 (CCNB1) to which p53 is directly bonded mediates G2/M progression and inhibits cell division. As reported, the inhibition of cyclin B and CDK1 led to the arrest of osteosarcoma cell division [34]. Inactivation of *CDK1* and *CDK2* triggered the apoptosis of osteosarcoma cells [35]. Moreover, MTX prevents tumor cells from proliferating by inhibiting dihydrofolate reductase (DHFR) [36]. The inhibition of *CDK* reduces the expression of both *DHFR* mRNA and protein thus enhancing sensitivity of human osteosarcoma cell lines to MTX [36, 37]. These studies concluded that the usage of combination of cyclin-CDK inhibitors and MTX which regulated mitotic cell cycle and p53 signaling pathway might overcome MTX resistance in osteosarcoma cells.

However, the DEGs identified in the present study were not the same with those identified to be associated with MTX resistance by Selga et al. using seven cell lines of different types of cancer [9]. This discrepancy may be attributed to the fact that genes identified by bioinformatics methods often vary with the criteria you adopt for analysis, and that Selga et al. focused on seeking genes commonly expressed in different MTX-resistant tumors, whereas we only paid attention to those specifically related to MTX resistance developed in osteosarcoma cell line. However, since there are only three samples for either MTX-sensitive or MTX-resistant cells, a very small sample, the universality and applicability of our findings is impaired, and further experimental proofs are needed to validate the findings.

## Conclusions

In conclusion, this study identified several potential molecular targets that might contribute to the MTX resistance in osteosarcoma cells, such as *GADD45A*, *AARS*, *AURKA*, *AURKB*, *CENPA*, *CCNB1*, *CCNE2*, and *CDK1*, which may function via aminoacyl-tRNA biosynthesis pathway, cell cycle pathway, or p53 signaling pathway. However, the finding here should be taken prudently.
